# Total skin electron therapy in the lying‐on‐the‐floor position using a customized flattening filter to accommodate frail patients

**DOI:** 10.1120/jacmp.v14i5.4309

**Published:** 2013-09-06

**Authors:** Christopher L. Deufel, John A. Antolak

**Affiliations:** ^1^ Department of Radiation Oncology Mayo Clinic Rochester MN USA

**Keywords:** cutaneous T‐cell lymphoma, flattening filter floor, Stanford, total skin electron

## Abstract

A total skin electron (TSE) floor technique is presented for treating patients who are unable to safely stand for extended durations. A customized flattening filter is used to eliminate the need for field junctioning, improve field uniformity, and reduce setup time. The flattening filter is constructed from copper and polycarbonate, fits into the linac's accessory slot, and is optimized to extend the useful height and width of the beam such that no field junctions are needed during treatment. A TSE floor with flattening filter (TSE FF) treatment course consisted of six patient positions: three supine and three prone. For all treatment fields, electron beam energy was 6 MeV; collimator settings were an x of 30 cm, y of 40 cm, and $\theta_{\text{coll}}$ of 0°; and a 0.4 cm thick polycarbonate spoiler was positioned in front of the patient. Percent depth dose (PDD) and photon contamination for the TSE FF technique were compared with our standard technique, which is similar to the Stanford technique. Beam profiles were measured using radiochromic film, and dose uniformity was verified using an anthropomorphic radiological phantom. The TSE FF technique met field uniformity requirements specified by the American Association of Physicists in Medicine Task Group 30. TSE FF $R_{80}$ ranges from 4 to 4.8 mm. TSE FF photon contamination was ~ 3%. Anthropomorphic radiological phantom verification demonstrated that dose to the entire skin surface was expected to be within about ±15% of the prescription dose, except for the perineum, scalp vertex, top of shoulder, and soles of the feet. The TSE floor technique presented herein eliminates field junctioning, is suitable for patients who cannot safely stand during treatment, and provides comparable quality and uniformity to the Stanford technique.

PACS number: 87

## I. INTRODUCTION

Total skin electron (TSE) therapy is used for the treatment of cutaneous T‐cell lymphoma, mycosis fungoides, and Kaposi sarcoma.^1^, ^2^, ^3^ TSE targets the entire skin to a penetration depth of several millimeters using 3 to 7 MeV electrons delivered with large fields and extended source to surface distance (SSD).^(3)^ A uniform dose to the entire skin surface is desired; however, skin crevices, surface curvature variations, and patient size create challenges in delivering the prescribed dose without regions of under‐ or overdosage.^4^, ^5^, ^6^ The Task Group 30 (TG30) of the American Association of Physicists in Medicine recommends that treatment field uniformity not exceed ±8% in the patient superior‐inferior direction and ±4% in the patient left‐right direction over the central $160\times 60\;{\text{cm}}^{2}$.^(3)^ However, *in vivo* dose to a patient is expectedly less homogeneous, and ±15% variation over the skin surface is typical, excepting certain areas such as perineum, axilla, and the scalp vertex.^(^ ^5^ ^,^ ^6^ ^)^

TSE dose is traditionally delivered using a Stanford six dual‐field or McGill rotational technique, with the majority of clinics preferring the Stanford technique.^(1)^ In this technique, a patient adopts six poses, incremented every 60°, through the course of treatment: anteroposterior (AP), posteroanterior (PA), right anterior oblique (RAO), right posterior oblique (RPO), left anterior oblique (LAO), and left posterior oblique (LPO).^(^ ^1^ ^,^ ^3^, ^4^, ^5^, ^6^, ^7^ ^)^ Each pose occurs behind a thin plastic scattering panel. Dual electron fields with central rays approximately ±20° from horizontal are delivered at extended SSD to provide large, uniform treatment fields. In the McGill technique, the patient is positioned on a motorized platform (rotating at three revolutions/minute) 3.8 m from the target of the linear accelerator (linac). The gantry is directed horizontally toward the patient and a customized flattening filter is mounted in the linac treatment accessory slot.^(8)^ The collimator jaws are set to $40\times 40\;{\text{cm}}^{2}$ and the collimator is rotated to 45° to provide the largest‐possible treatment field size. Beam quality and uniformity specifications for Stanford and McGill techniques can be found in TG30^(3)^ and Reynard et al.^(8)^

Stanford and McGill techniques require that patients remain standing for treatment durations of 10 to 30 minutes.^(^ ^1^ ^,^ ^8^ ^)^ For patients who are weakened, elderly, and nonambulatory, a six‐field floor technique was developed by Wu et al.^(9)^ Analogous to the Stanford technique, six dual fields are used and the electron beam is incident on the patient surface in rotational increments of 60°. Treatment in the AP and PA positions is delivered with the patient's umbilicus positioned directly below isocenter, the patient oriented head to foot perpendicular to the linac waveguide, and the use of gantry angles of ±25°. Treatment in the LPO, RPO, LAO, and RAO positions is delivered with oblique junction fields. The gantry is rotated to 60°, and the patient lies on the floor oriented head to foot parallel to the waveguide with the umbilicus ~ 220 cm lateral to isocenter. Field junctions are required for each oblique position because the field is insufficiently uniform at an SSD of 330 cm.

The approach presented herein is a hybrid of the McGill method and the method of Wu et al.,^(9)^ combining a flattening filter with lying on the floor treatment. Our clinic elected to develop a flattening filter to improve treatment field uniformity, eliminate the need for field junctioning, and reduce setup time. For AP and PA positions, the proximity of the floor (SSD ~ 2 m) lessened field uniformity such that the TG30 horizontal uniformity specification (±4% at 30 cm from central axis [CAX]) was not achieved with our clinic's open beam using the Wu technique. Our experience with setup time and personnel anxiety related to the type of TSE field junctioning used in the Wu study also led us to seek a method that did not require patient repositioning or match lines.

## II. MATERIALS AND METHODS

Measurements were performed using a Varian TrueBeam Linac (Varian Medical Systems Inc., Palo Alto, CA) with energy setting 6 MeV in HDTSE mode (2500 MU/min). For all TSE floor with flattening filter (TSE FF) treatment fields, the collimator settings were x equals 30 cm; y, 40 cm; and $\theta_{\text{coll}}$ equals 0°. A polycarbonate spoiler (2 m × 1 m × 4 mm) was used for electron scatter and beam energy degradation. For six dual‐field Stanford measurements, the collimator settings were x equals 40 cm; y, 40 cm; and $\theta_{\text{coll}}$ equals 0°. Gantry angles equaled 250° and 290°. The Stanford polycarbonate spoiler (2 m × 0.9 m × 6 mm) was positioned 212 cm lateral to isocenter (SSD = 322 cm to patient).

Radiochromic film (GAFCHROMIC EBT3; Lot# A05161201 Exp. May 2014; International Specialty Products Inc, Wayne, NJ) was used in uniformity, PDD, body factor, and anthropomorphic phantom measurements. Exposed EBT3 film was scanned on a flatbed scanner (Expression 10000XL; Epson America Inc, Long Beach, CA) in color mode (16 bits per red, green, or blue channel) with a resolution of 0.17 mm (150 dpi). Film was scanned in the central region of the scanner to reduce scanner location dependence. Marker dye corrections were performed according to the methods of McCaw et al.^(10)^ A film calibration curve was established using 0, 10, 25, 50, 100, 150, 200, 300, 400, 500, 750, 1000, 1500, and 2000 cGy delivered on films from the same box. Only the central 15 cm × 20 cm region of each EBT3 film sheet was used for measurement in order to assure adequate intersheet and intrasheet uniformity. EBT3 film uniformity within the central 15 × 20 cm region was evaluated for different regions within a sheet and sheets within a lot. The standard deviation of dose (200 cGy delivered) was < 1% within a sheet and 1.3% among different sheets.

### A. Patient treatment setup and calibration

**Figure 1:**
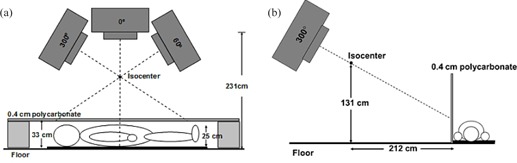
Total skin electron with flattening filter setup: (a) anteroposterior (AP)/posteroanterior (PA) treatment field; AP and PA positions were delivered with the patient's umbilicus positioned directly below isocenter, the patient oriented head to foot perpendicular to the linac waveguide, and gantry angles of 0°, 60°, and 300°; a 0.4 cm polycarbonate spoiler was supported 5 cm above the patient's proximal skin surface; ${\text{MU}}_{300{}^{\circ}}$ equals ${\text{MU}}_{60{}^{\circ}}$ and ${\text{MU}}_{0{}^{\circ}}$ equals $0.41\;{\text{MU}}_{60{}^{\circ}}$; and (b) the left posterior oblique, right posterior oblique, left anterior oblique, and right anterior oblique positions are delivered with the gantry rotated to 300°, the patient oriented head to foot parallel to the linac waveguide, and the umbilicus approximately 2.3 m lateral from isocenter. For all treatment fields, the linear accelerator (linac) mode was HDTSE 6 MeV; the collimator setting was x of 30 cm, y of 40 cm, and $\theta_{\text{coll}}$ of 0°; and a polycarbonate spoiler 0.4 cm thick was positioned in front of the patient.

AP and oblique patient setups are depicted in Fig. 1. The patient lies on a thin (3 cm) mat with arms and legs slightly away from the body and fingers spread apart.

AP and PA positions are set up with the patient's umbilicus positioned directly below isocenter, the patient oriented head to foot perpendicular to the linac waveguide, and the use of three gantry angles (0°, 60°, and 300°) to provide uniform treatment in the patient superior‐inferior direction. MU weighting for these gantry angles is the following: ${\text{MU}}_{300{}^{\circ}}$ equal to ${\text{MU}}_{60{}^{\circ}}$ and ${\text{MU}}_{0{}^{\circ}}$ equal to $0.41\;{\text{MU}}_{60{}^{\circ}}$. The collimator setting, gantry angles, and MU ratios were determined empirically. The polycarbonate spoiler is supported by foam blocks and is approximately 5 cm above the patient's proximal skin surface.

The LPO, RPO, LAO, and RAO positions are set up with the patient oriented in a head to foot direction parallel to the waveguide and the umbilicus 230 cm lateral to isocenter, with a gantry setting of 60°. As reported by Wu et al.,^(9)^ the dose to the scalp and soles of the feet may be enhanced by tilting the patient 5° from parallel. The polycarbonate spoiler is mounted upright with wooden supports and positioned immediately in front of the patient. Design specifications for the wooden supports are available on request from the authors.

Calibration of the TSE FF treatment resembles the methods used for the Stanford technique.^(3)^ A parallel plate ion chamber (Advanced Markus type No. TN34045; PTW, Freiburg, Germany) with electrometer (Model No. 616; Keithley Instruments Inc, Cleveland, OH) was used for calibrations in this study. Calibration factors (cGy/MU) refer to the surface of the patient, as opposed to $d_{\text{max}}$. First, a calibrated parallel plate chamber was positioned with its surface at the umbilicus treatment location, and TSE FF AP treatment field was delivered to obtain a cGy/MU calibration factor for the AP and PA fields. Next, the cGy/MU calibration factor for oblique treatment fields was determined by measuring cGy/MU at the RAO position at umbilical level from a single TSE FF oblique treatment field. Finally, an average body factor equal to 3.0 ± 0.1 was calculated by comparing dose from a single AP or oblique treatment field to the dose after all six fields have been delivered:

(1) $$\sum_{6\;Positions}Dose_{APPosition}=Dose_{APPosition}*BodyFactor$$

For body factor measurements, EBT3 film was affixed to the surface of RANDO (The Phantom Laboratory, Salem, NY) anthropomorphic phantom in 60° increments.

### B. Flattening filter design

**Figure 2:**
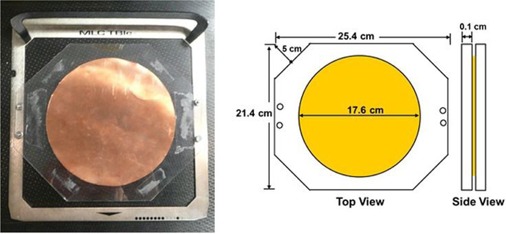
Customized flattening filter. A polycarbonate and copper flattening filter provides scatter and filtration for enhanced treatment field uniformity. A copper disc (diameter, 17.6 cm; thickness, 0.025 cm) is placed between two polycarbonate rectangles $\left(25.4\;\times \;21.4\;\times \;0.010\;{\text{cm}}^{3}\right)$. Corners of the polycarbonate have been trimmed 5 cm to increase transmission along the diagonals.

Figure 2 illustrates the customized flattening filter used in this study. The filter consists of a copper disc (diameter, 17.6 cm; thickness, 0.025 cm) interposed between two polycarbonate rectangles $\left(25.4\;\times \;21.4\;\times \;0.10\;{\text{cm}}^{3}\right)$. The filter was designed to use the same collimator settings for all treatment fields. Corners of the polycarbonate have been trimmed by 5 cm to increase transmission along the diagonals. The filter design is comparable to that of Reynard et al.^(8)^ Design differences include the use of copper instead of lead for hazardous materials safety reasons, elimination of the small aluminum disc for simplicity, reorientation of the filter for use with a 0° collimator setting, and adjustment of the dimensions for treatments at shorter SSDs.

Flattening filter design was determined empirically by optimizing superior‐inferior uniformity for the oblique treatment field with various filter dimensions. The variables tested included copper disc diameter, polycarbonate thickness, polycarbonate rectangle dimensions, and polycarbonate corner trimming. A more complicated filter design that incorporates additional materials or more thickness variations may be more efficient in producing a beam with equivalent uniformity. Separate filters for AP and oblique treatment fields would also be more efficient by accommodating the different electron scattering powers needed for their respective effective SSDs. Nevertheless, a single filter with simple design was chosen for our clinic because of the ease in manufacturing and for patient safety. More than one filter would allow for the possible use of the wrong filter for a given treatment field.

**Figure 3:**
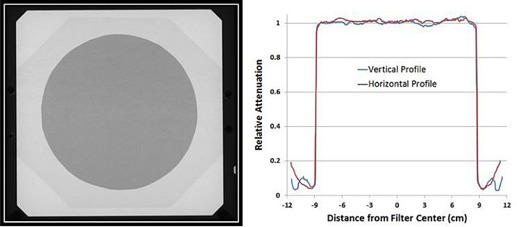
Simulator image of the flattening filter for verifying material uniformity. The flattening filter was imaged with a Varian iX simulator at 125 kVp. Vertical and horizontal profiles are shown.

Material uniformity (copper and polycarbonate) in the filter was verified by imaging the filter at 125 kVp using a Varian Acuity iX simulator (Varian Medical Systems). Figure 3 presents the digital image along with median‐filtered vertical and horizontal attenuation profiles.

### C. Treatment field uniformity

Treatment field uniformity was evaluated by affixing 2.5 cm × 2.5 cm EBT3 film squares to the polycarbonate spoiler surface. Approximately 200 cGy was delivered to the films, and dose was normalized to the location of the umbilicus. (Please refer to Materials & Methods Section A above for a description of TSE FF spoiler locations and dose delivery.) In the Stanford technique, the 0.6 cm polycarbonate spoiler was located 212 cm lateral to isocenter, and six dual fields (250° and 290° gantry angles) were delivered.

### D. Percent depth dose

Percent depth dose (PDD) curves for the HDTSE beam were measured with EBT3 film interposed between solid water or phantom slices, and irradiated edge‐on to deliver a maximum dose of 300 cGy. Care was taken to assure that film was aligned with the phantom surface and no air gaps were present.

A PDD at 100 cm SSD with $10\times 10\;{\text{cm}}^{2}$ applicator and no spoiler was measured in solid water. Six‐field PDDs for Stanford and TSE FF were measured in RANDO anthropomorphic phantom. The exact shape and size of the phantom is not expected to have a significant effect on six‐field PDD results.^(6)^

In the Stanford technique, RANDO phantom was placed at umbilical level with SSD equal to 322 cm. A 0.6 cm polycarbonate spoiler was located 10 cm in front of the phantom. Six dual fields (250° and 290° gantry angles) were delivered with the phantom rotated 60° after each dual field.

PDD for the TSE FF technique was measured at two anatomic locations (0 cm superior‐inferior and 80 cm superior of umbilicus), since positions superior and inferior to the umbilicus receive a less penetrating dose because of higher angles of incidence. TSE FF beam delivery is described in the Materials & Methods Section A above.

### E. Photon contamination

Photon contamination for AP and oblique setups was reported as the dose at 5 cm depth in solid water relative to the surface dose, measured at umbilical level. Photon contamination was measured in two ways: 1) directly from six‐field PDD curves in RANDO anthropomorphic phantom, and 2) using an Advanced Markus parallel plate ion chamber (PTW). Two methods were employed in order to confirm the accuracy of EBT3 film in the low dose region.

Using a parallel plate ion chamber, six‐field photon contamination is calculated after measuring per‐field photon contamination. Per‐field photon contamination, $\gamma_{\left(1‐\text{field}\right)}$, may be

(2) $$\gamma_{1-field}=\frac{D(d=5)}{D(d=0)}$$

Since surface dose at normal incidence is identical for all six treatment fields: $D_{\text{AP}}(d\;=\;0)=\;D_{\text{Oblique}}\left(d\;=\;0\right)$, cumulative photon contamination, $\gamma_{6‐\text{field}}$, may be expressed as:

(3) $$\gamma_{6-field}=\frac{\sum_{Fields}D(d=5)}{\sum_{Fields}D(d=0)}=\frac{2D_{AP}(d=5)+4D_{Oblique}(d=5)}{Bod\text{yF}actor*D_{AP}(d=0)}=\frac{\sum_{Fields}\gamma_{1-field}}{Bod\text{yF}actor}$$

Percent depth ionization was obtained using a minimum 15 cm of solid water placed beneath the ion chamber to provide adequate backscatter. Percent depth ionization was converted to PDD following the method of Ding et al.^(11)^ In their method, negligible differences in $P_{\text{wall}}$ and $P_{\text{Repl}}$ between electrons and photons are ignored:

(4) $$\gamma_{1-field}=\frac{D(d=5)}{D(d=0)}≅\frac{I(d=5)}{I(d=0)}\times \frac{{\left(\frac{L}{\rho }\right)}_{air}^{water}|{\;}_{d=R_{p}}}{{\left(\frac{L}{\rho }\right)}_{air}^{water}|{\;}_{d=0}}$$

As recommended by AAPM Task Group 70, stopping power ratios were calculated using the equation of Burns et al.^(12)^ Beam parameters $R_{p}$ and $R_{50}$ for TSE FF and Stanford techniques were collected from EBT3 film PDDs, where a single normal incidence beam (i.e., not a composite of multiple oblique beams) was delivered to the phantom.

### F. Anthropomorphic radiological phantom verification

The complete six‐position TSE FF was administered to a PIXY full‐body radiological phantom (Supertech Inc, Elkhart, IN) in order to assess uniformity of coverage and identify areas of over‐ and underdose. Dose at the skin surface was measured using EBT3 film. Phantom setup was the same as the treatment setup presented in Material & Methods Section A above. As described above, the phantom was tilted approximately 5° for oblique beams to provide greater dose to the scalp and soles of the feet.

## III. RESULTS

### A. Treatment field uniformity

**Figure 4:**
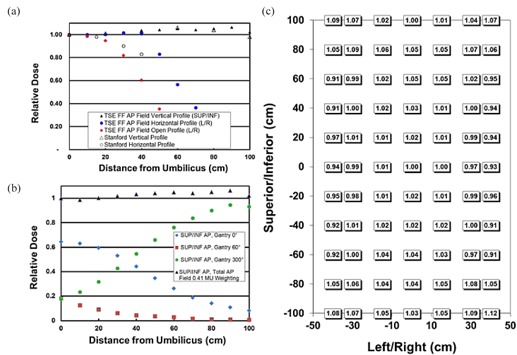
AP/PA treatment field uniformity measured with radiochromic film. Film was affixed to the spoiler surface (SSD = 206 cm). MU weighting for the gantry angles is ${\text{MU}}_{0{}^{\circ}}\;=\;0.41\;{\text{MU}}_{60{}^{\circ}}$. Vertical (superior/inferior [SUP/INF]) and horizontal (left/right [L/R]) profiles (a) drawn through the umbilicus region; the open horizontal profile is obtained without the flattening filter and with a collimator setting of $40\times 40\;{\text{cm}}^{2}$ and $\theta_{\text{coll}}$ equals 0°; Stanford technique profiles are provided for comparison. SUP/INF AP profiles (b) for individual and combined gantry angles; the combined AP SUP/INF profile used ${\text{MU}}_{300{}^{\circ}}$ equals ${\text{MU}}_{60{}^{\circ}}$ and ${\text{MU}}_{0{}^{\circ}}\;=\;0.41\;{\text{MU}}_{60{}^{\circ}}$. TSE FF AP field measurements (c) over an area of $200\times 80\;{\text{cm}}^{2}$. AP/PA indicates anteroposterior/posteroanterior.

**Figure 5:**
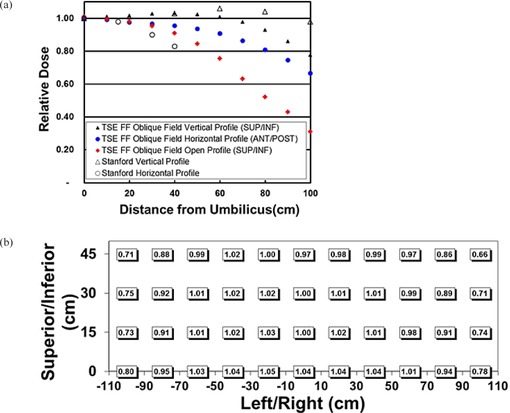
Oblique treatment field uniformity measured with radiochromic film. Film was affixed to the spoiler surface 212 cm lateral to isocenter. The gantry angle equals 300°. Vertical (superior/inferior [SUP/INF]) and horizontal (anterior/posterior [ANT/POST]) profiles (a) were drawn through the umbilicus region; the open vertical profile was obtained without the flattening filter and with a collimator setting of $40\times 40\;{\text{cm}}^{2}$ and $\theta_{\text{coll}}$ equals 0°; Stanford technique profiles are provided for comparison. TSE FF oblique field measurements (b) over an area of $200\times 45\;{\text{cm}}^{2}$.

Figures 4 and 5 summarize treatment field uniformity results for the TSE FF technique. Stanford technique profiles measured in our clinic are provided for comparison. Vertical and horizontal profiles (Fig. 4(a) and Fig. 5(a)) are drawn through the umbilicus. Open field profiles without the customized flattening filter were also measured to evaluate the impact of the filter. Open field measurements used a collimator setting of 9 equals 0° and $40\times 40\;{\text{cm}}^{2}$ with a gantry setting of 0°.

AP treatment field uniformity results (Fig. 4) demonstrate vertical (superior‐inferior) uniformity ±5% up to 100 cm from umbilicus and horizontal (left‐right) uniformity ±4% up to 35 cm from umbilicus. The open field (customized flattening filter removed) profile along the left‐right direction is notably less uniform than the same profile using the filter, with dose falling to 82% by 30 cm. Empirical determination of relative MU weighting for 0°, 60°, and 300° gantry angles is illustrated in Fig. 4(b). MU weighting ${\text{MU}}_{300{}^{\circ}}$ equal to ${\text{MU}}_{60{}^{\circ}}$ and ${\text{MU}}_{0{}^{\circ}}$ equal to 0.41 ${\text{MU}}_{60{}^{\circ}}$ produced a combined AP profile with acceptable superior‐inferior uniformity. The range of doses for the AP field is presented in Fig. 4(c).

Oblique treatment field uniformity results (Fig. 5) demonstrate vertical uniformity of ±8% up to 80 cm from umbilicus and horizontal uniformity of ±4% up to 40 cm from umbilicus. The open field profile along the superior‐inferior direction is provided for comparison. The range of doses for the oblique field is presented in Fig. 5(b), where the anterior‐posterior axis equals zero at the floor level. Figure 5(a) (open profile vs. vertical profile) illustrates how the flattening filter eliminates field junctions for oblique fields. Without the filter (open profile) the dose falls below 90% approximately 40 cm from umbilicus.

### B. Percent depth dose

**Figure 6:**
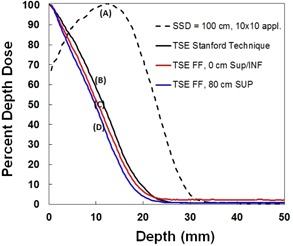
Percent depth dose (PDD) measured with radiochromic film. PDD (a) for the 6 MeV HDTSE beam at 100 cm SSD with a standard $10\times 10\;{\text{cm}}^{2}$ applicator; TSE PDD (b) for the six dual‐field Stanford technique at 322 cm SSD with a collimator setting of $40\times 40\;{\text{cm}}^{2}$; PDD at umbilical level (c) for the TSE FF technique with the custom flattening filter; PDD 80 cm superior to the umbilicus (d) for the TSE FF technique. Error bars representing one standard deviation on film measurements are approximately 3% (n=6). SSD indicates source to surface distance; TSE FF stands for total skin electron floor with flattening filter.

PDD measurements are summarized in Fig. 6. The TSE FF and TSE Stanford curves represent the average PDD over various regions of the phantom. Error bars representing one standard deviation on film measurements are approximately 3% (n=6).

TSE FF PDD at umbilical level (body) is similar to the Stanford technique, with $R_{80}$ for the two techniques equal to 4.8 and 5.8 mm, respectively. TSE FF PDD at the level of +80 cm (head) is less penetrating, with an $R_{80}$ equal to 4 mm. Additional beam quality parameters may be found in Table 1. $R_{50},d_{\text{max}}$, and $R_{p}$ were measured from data presented in Fig. 6. $E_{0}$ was calculated from $R_{50}$ according to TG70 recommendations.^(13)^

**Table 1:** Treatment beam quality parameters

*Treatment Technique, Field*	*R50 (cm)* ^a^	$d_{\text{max}}\;(\text{cm})$ ^a^	${Ē}_{0}\;(\text{MeV})$ ^b^	$R^{p}\;(\text{cm})$ ^a^
TSE FF, 6‐Field Head	0.9	0	2.5	1.9
TSE FF, 6‐Field Body	1.0	0	2.7	2.0
Stanford 6‐Field	1.1	0	2.9	2.0
6 MeV, SSD = 100 cm	2.2	1.3	5.3	2.9

a Measured from Figure 6.

b Calculated using TG70: ${Ē}_{0}\;=\;0.656\;+\;2.059R_{50}\;+\;{0.022R}_{50}^{2}$.

### C. Photon contamination

Per field and cumulative photon contamination results are provided in Table 2. EBT3 film and ionization chamber results show good agreement. The TSE FF technique introduces greater photon contamination (~ 3%) than the Stanford technique (~ 0.6%) because of bremsstrahlung from the flattening filter, consistent with the results of Reynard et al.^(8)^ The oblique field has slightly greater contamination than the AP field (see the Discussion Section below).

**Table 2:** TSE photon contamination measurements

	*Ionization Chamber*				*Radiochromic Film*
*Treatment Technique, field*	*Measured PDI Ratio PP Chamber, %*	*γ*	*γ Contamination, Single field, %*	*γ Contamination, Cumulative 6‐field, %*	*γ Contamination, Cumulative 6‐field, %*
TSE FF, anteroposterior	1.4	1.08	1.5	3.4	2.5
TSE FF, oblique	1.7	1.08	1.8		
TSE Stanford, anteroposterior	0.2	1.08	0.2	0.4	0.7

FF = floor with flattening filter; PDI = percent depth ionization; PP = parallel plate; TSE = total skin electron.

### D. Anthropomorphic radiological phantom

Film results for the PIXY phantom are presented in Table 3. Doses at various anatomic locations are typically within ±15% of prescription dose, with exceptions that include soles of the feet, top of shoulder, vertex of the scalp, and medial thigh and perineum regions. Variation in dose for the PIXY phantom is comparable to patient TLD measurements reported by Antolak et al.^(6)^ using the Stanford technique.

**Table 3:** Anthropomorphic phantom verification of TSE FF clinical treatment using PIXY phantom with radiochromic film

	*Normalized Dose*	
*Anatomic Site*	*TSE FF, PIXY, %*	*Stanford, Antolak et al*.,^(6)^ %^a^
Umbilicus, anterior	96	100±4
Umbilicus, lateral	96	98±6
Back, upper	99	93±7
Back, lower	101	91±7
Thorax, upper	95	93±4
Thigh, midanterior	99	100±9
Buttock	97	58±14
Elbow, posterior	98	90±13
Forehead	104	96±8
Shoulder, lateral	107	100±12
Scalp	106	105±8
Submental	114	101±6
Foot, mid‐dorsum	112	117±7
Toe, middle anterior	112	141±10
Axilla	85	60±25
Hand, dorsum	84	85±6
Finger, midmedial	80	123±27
Scalp, vertex	63	87±20
Shoulder, top	62	74±8
Thigh, upper medial	60	54±25
Perineum	41	25±21
Foot, sole	4	N/A

a Stanford technique patient TLD data: dose error is reported as 1 sample SD

FF = floor with flattening filter; TSE = total skin electron.

## IV. DISCUSSION

Table 4 summarizes treatment time, beam quality, and uniformity for the TSE FF and Stanford techniques used in our clinic. The Stanford technique continues to be the standard of care for stable ambulatory patients in our practice. The TSE FF technique is reserved for those patients who cannot safely stand for the duration of the Stanford technique. The lying‐on‐the‐floor positions in the TSE FF technique may not afford the same degree of coverage as the Stanford technique in the groin, perineum, and buttocks and in the underarm for obese patients because of skin touching, but this effect can be partially alleviated with appropriate boost fields. Additional care should be taken during the TSE FF patient setup to maintain the patient's arms away from the sides, with fingers and legs spread apart.

Fingernail and eye shields should be considered for all patients and discussed with the physician. Furthermore, the patient's hands are expected to receive prescription dose since the hands are positioned at the patient's side and not holding supports, as in the Stanford and McGill techniques. This position is an advantage for patients who have substantial disease on the hands; however, shielding of the hands for a portion of the treatment may be considered if the patient has uncomfortable redness or swelling, or both.

**Table 4:** Comparison of treatment parameters for the TSE FF and Stanford techniques

	*TSE FF*	*Stanford*
Treatment “Beam‐On” Time $\left({\text{MU}}_{6\text{field}/\text{Gy}}\right)$	8820	5672
6‐Field Characteristics		
$R_{80}\;(\text{mm})$	4.8	5.8
$R_{50}\;(\text{mm})$	10	11
γContamination (%)	~ 3	~ 0.6
Vertical Uniformity, 90% width (cm)		
AP Field (SUP/INF)	>200	
Oblique Field (SUP/INF)	160	
Dual Field (SUP/INF)		>200
Horizontal Uniformity, 90% width (cm)		
AP Field (L/R)	90	
Oblique Field (ANT/POST)	60	
Dual Field (L/R)		60

Anthropomorphic phantom results provide an estimate of anatomic locations that may be over‐ or underdosed (Table 3). It has been strongly recommended that *in vivo* dosimetry be performed on all patients;^(^ ^3^ ^,^ ^6^ ^)^ therefore, diodes, TLDs, or a comparable dosimeter should be placed on each patient for the first few sessions to assess any differences that arise as a result of patient‐specific anatomy.

For patients taller or wider than 160 cm × 60 cm, modifications to the treatment may need to be made. For tall patients, the SSD of the oblique treatment field may need to be increased and calibration adjusted. Uniformity over the patient's circumference should not change significantly as long as CAX still intersects the patient at ~ 60° angle of incidence for oblique fields. For wide patients, hands and forearms are the typical regions extending laterally beyond 30 cm. Figures 4 and 5 demonstrate that lateral uniformity should be adequate up to 40 cm for hands and forearms. If additional uniformity is necessary, hands or forearms may be elevated to bring them closer to the spoiler.

TSE FF PDDs are slightly less penetrating than the Stanford PDD. Such differences in penetration should be discussed with the physician before treatment, particularly if the patient has disease of unusual thickness near the head or foot regions of the body. TSE FF $R_{80}$ is ~ 1 mm shallower near head and foot levels than at umbilicus level.

PIXY doses to the scalp vertex and soles of the feet were lower than measurements obtained by Wu et al.^(9)^ The dose difference is in part due to a vertical uniformity difference. A thicker spoiler would improve uniformity, but was not used here because it would reduce penetration of the beam to less than what is clinically desired. Another reason for the dose difference is the absence of field junctions. With oblique field junctions, feet are treated separately from the head and may be always tilted towards the beam. Thus, the benefit from tilting the patient 5° is expected to be only half that of the method used by Wu and colleagues, and boosts to the scalp and sole of the foot may be larger using the TSE FF technique.

The oblique field has slightly greater photon contamination than the AP field due to differences between effective photon and electrons source to surface distances: ${\text{SSD}}_{\text{eff}e‐},\;{\text{SSD}}_{\text{eff}\gamma }$ The effective electron source may be estimated from dose per monitor unit at phantom surface as a function of distance, and is located slightly upstream of the accessory tray/flattening filter. The effective photon source may be similarly estimated using dose per monitor unit at 5 cm depth in phantom, and is located further upstream from the flattening filter. ${\text{SSD}}_{\text{eff}\;e‐}$ is less than ${\text{SSD}}_{\text{eff}\;\gamma }$, and, therefore, electron dose falls off faster than photon dose as a function of distance. Thus, the ratio of photon to electron dose increases as a function of SSD for TSE FF. Equations describing how the effective source position changes for TSE beam geometry have been derived by Antolak and Hogstrom.^(14)^

Quality assurance for the TSE FF technique follows Stanford technique TG30 recommendations. Of course, each clinic should establish a quality assurance schedule that best reflects the institution's clinical application, capabilities, and caseload.

## V. CONCLUSIONS

TSE FF using a customized flattening filter provides a suitable alternative to the Stanford technique for nonambulatory patients. TSE FF meets the irradiation field requirements specified by the American Association of Physicists in Medicine TG30 of ±8% vertical uniformity and ±4% horizontal uniformity over the central $160\times 60\;{\text{cm}}^{2}$, and comprehensive treatment to a radiological phantom demonstrates acceptable uniformity for treatment, with a potential need for electron boosts to the soles of the feet, perineum region, and scalp vertex.

## ACKNOWLEDGMENTS

The authors thank Terrance A. Harms, Robert W. Kline, and Luis E. Fong de los Santos for their suggestions and discussion throughout this project's development.

## Supporting information

Supplementary Material
