# Supplementary figures and images for: Total skin electron therapy in the lying‐on‐the‐floor position using a customized flattening filter to accommodate frail patients
Source: J Appl Clin Med Phys. 2013 Sep 6;14(5):115–26. doi: 10.1120/jacmp.v14i5.4309 (PMC5714577; doi:10.1120/jacmp.v14i5.4309)

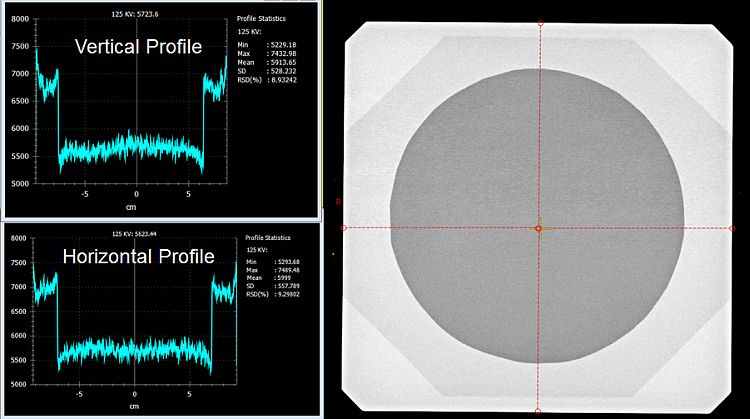

Supplement: Supplementary file 1 — Supplementary Material [file ACM2-14-115-s001.jpg]
